# Implicit 3D geological modeling method based on expert knowledge constraints: A case study from the Songshugang Mining District, Hengfeng County, Jiangxi Province, China

**DOI:** 10.1371/journal.pone.0331937

**Published:** 2026-05-18

**Authors:** Wenlong Jin, Zhichun Wu, Zhe Xu, Hongda Li, Bin Li, Fusheng Guo, Zhiqiang Zhang, Fenling Ma, Hualiang Li, Peng Lu

**Affiliations:** 1 School of Earth and Planetary Sciences, East China University of Technology, Nanchang, China; 2 Basic Geological Survey Institute, Jiangxi Geological Survey and Exploration Institute, Nanchang, China; 3 Radioactive Resources and Environmental Survey Center, Zhejiang Province Nuclear Industry 262 Brigade, Huzhou, China; 4 Geological Exploration Institute, Shanxi Geological and Mineral Third Team Co., Ltd, Baoji, China; Ural Federal University named after the first President of Russia B N Yeltsin: Ural’skij federal’nyj universitet imeni pervogo Prezidenta Rossii B N El’cina, RUSSIAN FEDERATION

## Abstract

Implicit 3D geological modeling offers advantages in rapid model construction; however, purely data-driven approaches have limitations in modeling accuracy and often fail to represent complex geological structures faithfully. To enhance the accuracy and applicability of implicit 3D geological modeling, a method that integrates data-driven approaches with expert knowledge constraints is proposed. Using Leapfrog Geo as the platform, this study integrated multi-source data (boreholes, exploration line profiles) with geological expertise and a priori constraints, to achieve a high-precision reconstruction of the complex geological structure and 3D grade distribution in the Songshugang Ta-Nb deposit, Hengfeng, Jiangxi. Subsequently, deep-seated mineralization targets were predicted. The results demonstrate that this method significantly enhances the modeling accuracy and efficiency for intricate geological bodies. The constructed three-dimensional geological model indicates that the mineralization enrichment zones are located at the contact zones between sodic feldspar granite and the overlying potassic feldspar granite and greisenized granite, as well as between sodic feldspar granite and the underlying biotite granite. Based on this, two favorable mineral exploration targets have been predicted in the deeper regions.

## 1. Introduction

Three-dimensional (3D) geological modeling enables the intuitive and accurate visualization of subsurface geological structures, providing critical support for mineralization prediction, resource exploration, and engineering design [1–3]. Explicit 3D geological modeling methods, which depend heavily on manual interpretation, suffer from limitations including low efficiency, a strong multi-solution nature, and difficulty in reconstructing complex geological interfaces [4–8]. Implicit 3D geological modeling involves the parametric representation of 3D geological interfaces using mathematical functions. This approach not only enables the automation of the modeling process but also allows for the precise expression of the spatial continuity of complex geological interfaces, thereby significantly enhancing modeling efficiency [9–13]. Compared to explicit 3D geological modeling, implicit 3D geological modeling has two significant advantages. First, for data integration, spatial interpolation algorithms synthesize diverse sources-including geological maps, cross-sections, and borehole logs-to establish a unified mathematical representation of geological information [3,14–19]; second, in terms of modeling efficiency, implicit 3D geological modeling generates 3D geological models directly from scattered points through function space interpolation, significantly improving modeling speed and enabling rapid model updates in response to changes in modeling data [20–24].

The spatial distribution patterns and 3D geometries of geological bodies are governed by the superposition and interaction of multistage geological processes-including tectonic deformation, sedimentation, metamorphic reworking, and magmatic intrusions-resulting in pronounced spatial heterogeneity, morphological complexity, and multi-scale nested features. For such complex geological bodies, an exclusive reliance on mathematical interpolation methods frequently fails to incorporate geological genetic constraints adequately, consequently introducing systematic deviations between modeling results and observed geological conditions. Particularly under conditions of sparse data and complex geological architectures, purely data-driven implicit 3D geological modeling struggles to resolve intricate subsurface geometries accurately. This frequently results in compromised model accuracy and may even preclude complete model generation [10,13,16,25,26]. Consequently, prior research on implicit 3D geological modeling has predominantly focused on reconstructing structurally simple geological bodies within data-rich environments [27–28].

Based on the cognitive framework of modelers regarding the deep geological structure of the modeling area, a modeling method that integrates data-driven approaches with expert knowledge constraints is employed. This method transforms expert knowledge into implicit constraints for three-dimensional geological modeling. This expert knowledge-integrated approach effectively reduces solution ambiguity, enhances modeling precision and credibility, and ensures that the resultant 3D models conform to regional geological principles and structural evolution history. This study investigates expert knowledge-constrained implicit 3D geological modeling methodologies through a case study of the Songshugang mining district, Hengfeng County, Jiangxi Province. By integrating multi-source data-including borehole logs and exploration cross-sections-within the Leapfrog Geo platform, we construct comprehensive 3D geological framework and grade models. This foundation enables prediction of deep subsurface mineralization targets, providing critical guidance for subsequent exploration targeting.

## 2. Regional geological setting

### 2.1. Regional geological characteristics

The Songshugang mining district occupies Geyuan Town, Hengfeng County, Jiangxi Province. Positioned approximately 3 km northwest of the Lingshan Pluton contact zone, it spans coordinates 117°37’28”E-117°38’58”E and 28°37’15”N-28°38’30”N. Tectonically, the mining district occupies a critical position within the suture zone between the Yangtze Block and Cathaysia Block; metallogenically, it lies within the Huaiyu Mountains Metallogenic Subzone of the Qinzhou-Hangzhou Metallogenic Belt’s eastern segment [29].

**Fig 1:**
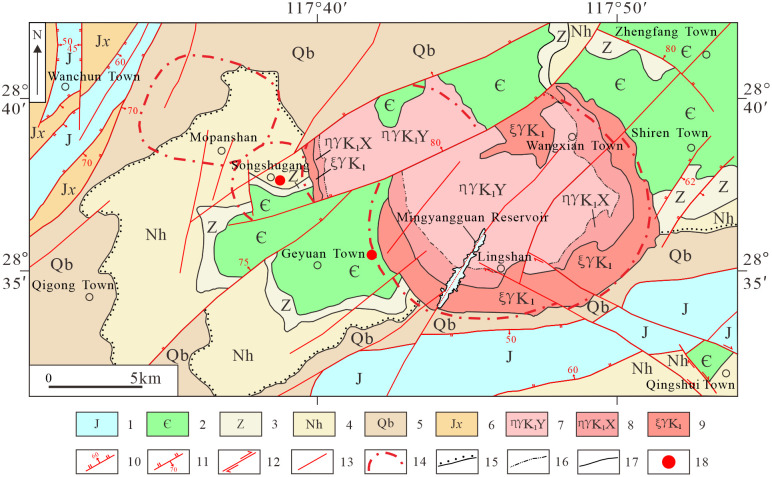
Regional geological map (modified from [32]). 1. Jurassic System; 2. Cambrian System; 3. Sinian System; 4. Nanhua System; 5. Qingbaikouan System; 6. Jixian System; 7. Yangqiao Unit; 8. Xiazhaiwu Unit; 9. Lingshan Superunit; 10. Normal fault, dip angle; 11. Reverse fault, dip angle; 12. Strike-slip fault; 13. Fault (nature undefined); 14. Circular structure (RS-interpreted); 15. Unconformity contact; 16. Lithofacies boundary; 17. Geological contact; 18. Tantalum-niobium deposit.

The region primarily exposes strata of the Jixian, Qingbaikou, Nanhua, Sinian, Cambrian, and Jurassic Systems (Fig 1). The Jixian System is composed predominantly of phyllites and schists; the Qingbaikouan System comprises phyllites, rhyolites, and metaclastic lithic conglomerates; the Nanhua System comprises phyllites, slates, pebbly greywackes, tuffites, and carbonaceous shales; the Sinian System comprises siltstones and fine-grained sandstones; the Cambrian System consists of calcareous mudstones and argillaceous limestones; the Jurassic System includes mudstones, conglomeratic sandstones, and siltstones. Magmatic rocks are dominated by the Lingshan composite batholith, exhibiting distinct lithofacies zoning. Three concentric zones are identified from core to periphery: Fine-grained glomeroporphyritic rapakivi amphibole-biotite monzogranite, Medium-grained rapakivi amphibole-biotite monzogranite, Medium-coarse-grained biotite alkali-feldspar granite [30].

The area exhibits well-developed NE-SW trending linear structures, with subordinate NW-SE orientations, with subordinate NW-SE trends. Sub-EW trending basin-controlling faults occur along the southern margin of the Jurassic basin. Under the influence of the Lingshan batholith and deep-seated concealed intrusions, three distinct circular structures-Mopanshan, Songshugang, and Lingshan-are manifested on remote sensing imagery. Proven deposits include the Songshugang Ta-Nb and Huangshan Nb-Ta deposits. Ore bodies are predominantly occur within altered granitic intrusions, comprising potassic-altered granite, sodic-altered granite, and greisenized granite types. Surface country rocks exhibit intense alteration assemblages featuring potassic alteration, albitization, greisenization, hematitization, sericitization, and chloritization [31].

Reprinted from [32] under a CC BY license, with permission from [Geocarto International], original copyright [2025].

### 2.2. Geological setting of the mining district

**Fig 2:**
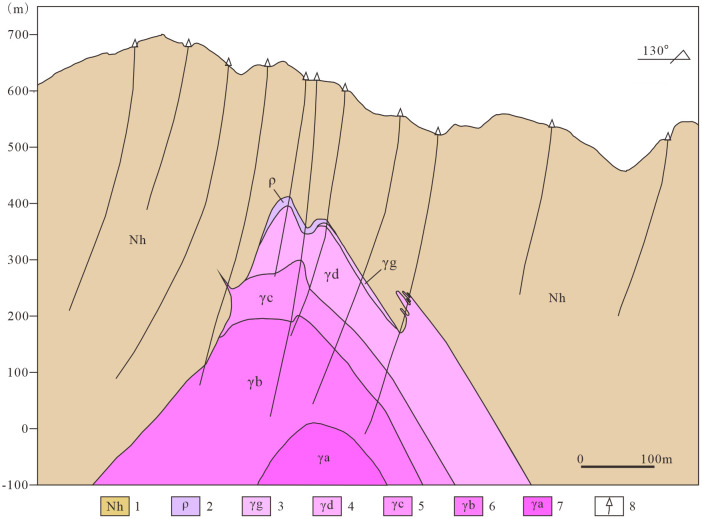
Section along exploration line 0 in the songshugang mining area, Hengfeng County, Jiangxi Province. 1. Nanhua System; 2. Pegmatite-type Nb-Ta mineralized zone; 3. Greisenized granite-type Nb-Ta mineralized zone; 4. K-feldspathized granite-type Nb-Ta mineralized zone; 5. Weak albitized granite subzone; 6. Moderate albitized granite subzone; 7. Strong albitized granite subzone; 8. Boreholes.

The Songshugang mining district constitutes a concealed, altered granite-hosted rare-metals deposit. Tantalum (Ta) and niobium (Nb) represent the primary economic metals, with associated mineralization including tungsten (W), tin (Sn), rubidium (Rb), and lithium (Li). This deposit exhibits distinct metallogenic characteristics, with ore bodies occurring within alteration shells atop concealed granite cupolas. This mineralization pattern features spatially coexistent and genetically associated polymetallic assemblages. Surface exposures comprise the Nanhua System, dominated by low-grade metamorphic phyllites and slates. The deep-seated concealed rock bodies associated with mineralization are divided into six alteration zones: pegmatite-type niobium-tantalum mineralization zone, greisenized granite-type niobium-tantalum mineralization zone, potassic feldsparized granite-type niobium-tantalum mineralization zone, weakly albitized granite-type subzone, moderately albitized granite-type subzone, and strongly albitized granite-type subzone (Fig 2) [33]. The mineralogical characteristics of each alteration zone are presented in Table 1. The wall-rock alteration shows a vertical zonation. From the bottom up, there are a sodic alteration zone, a greisen zone, and a potassic alteration zone, which have a close spatial and genetic relationship with mineralization. In terms of structure, the mining district is on the northern limb of the Geyuan-Zhengfang composite syncline, with many secondary folds. The fault structures in the district are mainly small-scale NE and NW-trending faults [31].

**Table 1:** Mineral Characteristics of Rock Alteration Zones.

Alteration zoning in rock masses		Mineral characteristics
Pegmatite-type niobium-tantalum mineralization zone (ρ)		The mineralized rock is a pegmatite, composed of coarse-grained flesh-red microcline (70–80%), quartz (10–15%), lepidolite (5–8%), and albite. The grain size of the minerals is relatively uniform, generally ranging from 5 to 7 millimeters, with some grains reaching up to 15 millimeters.
Potassic feldspar-altered granite-type niobium-tantalum mineralization zone (γd)		The mineralized rock is potassic granite, almost entirely composed of flesh-red euhedral to subhedral fine-grained microcline (over 90%), with small mineral grains ranging in size from 1.3 to 3 millimeters.
Greisenized granite-hosted niobium-tantalum mineralization zone (γg)		The mineralized rock is a potassic (albitized) greisenized granite, with locally strong greisenization forming greisen. The rock is gray, light red, or gray-green in color, with a scaly metamorphic texture, granitic relict texture, and porphyroblastic texture, and a massive structure. Microcline feldspar often appears as porphyroblasts with a grain size of 1–3 mm, while quartz has a grain size of 3–5 mm. The rock is primarily composed of quartz (45–50%), lepidolite (15–25%), topaz (10–15%), and microcline feldspar (10–15%), with minor amounts of sericite and fluorite.
Sodium feldspar-altered granite-type niobium-tantalum mineralization zone.	Weakly sodic granitic subzone (γc)	The mineral composition is primarily composed of microcline, quartz, albite, and lepidolite, with respective contents of 40–43%, 35%, 20–26%, and 3–5% of the whole rock.
	Moderately sodic granite subzone (γb)	The primary minerals are microcline (35–45%), quartz (25–30%), albite (20–30%), and lepidolite (3–7%), with secondary minerals including topaz.
	Strongly sodic granitic subzone (γa)	The rock is composed of albite (25–40%), microcline (20–30%), quartz (15–25%), and lepidolite (3–6%), with secondary amounts of topaz.

## 3. FastRBF function introduction and modeling workflow

### 3.1. FastRBF introduction

Radial Basis Function (RBF) has become one of the most widely used functions in implicit 3D geological modeling due to its mathematical flexibility, accurate characterization of complex spatial relationships, and strong adaptability to unstructured geological data. In contrast to traditional interpolation methods like Kriging and Discrete Smooth Interpolation (DSI), RBF transforms spatial scatter points into a continuous implicit field by employing kernel functions. This capability not only facilitates the effective representation of nonlinear geometric features inherent in complex geological interfaces but also accommodates sparse and heterogeneously distributed geological data with remarkable efficiency.

RBF modeling generates a continuous, implicit surface from scattered data points. The model uses inputs like point coordinates, normal vectors, and other constraints to calculate the weighting coefficients for the basis functions. By incorporating global polynomial terms to correct low-frequency errors, this method produces a smooth, accurate implicit surface equation [20,34,35,36,37]. Obtain the zero isosurface *F*(*r*)=0, constitutes the surface of the geological body model, with the implicit surface equation constructed from scattered data points [38]:


$$F(r)=\sum_{i=1}^{N}w_{i}\phi \left(∥r-q_{i}∥\right)+P(r)=0$$ (1)

In the formula: *r* is any data point on the generated surface; *q*_*i*_ are the control points (known sample points, i.e., surface points); *w*_*i*_ are the weights corresponding to each control point; $\phi \left(∥r-q_{i}∥\right)$ is the radial basis function; and *P*(*r*) is a first-order polynomial in *r*.


$$P(r)=a_{0}+a_{1}x+a_{2}y+a_{3}z$$ (2)

The kernel function type of the FastRBF function is the improved spheroidal basis function, and the specific formula is:


$$\phi (c,m,r)=\left\{ \begin{array}{c} 1-\frac{r}{c}\frac{m}{\sqrt{1+m}}\\ \frac{1}{2}(\frac{m+1}{m+2}{)}^{-1-\frac{m}{2}}{\left(1+(\frac{r}{c}{)}^{2}\right)}^{-\frac{m}{2}} \end{array}\begin{array}{c} <\frac{c}{\sqrt{m+1}}\\ r>\frac{c}{\sqrt{m+1}} \end{array}\right.$$ (3)

In the formula: *r* is the distance between the calculation point and the center of the basis function; *m* is the order of the ellipsoidal function (an odd number between 3 and 9), which controls the rate of decay of the function, with smaller *m* values resulting in slower decay and smoother functions, and larger *m* values resulting in faster decay and more localized functions; *c* is the parameter that controls the “support radius” of the kernel function; this formula is a decreasing function that asymptotically approaches zero.

In Leapfrog software, the basis functions are represented in the form of spherical variograms:


$$\gamma (r)=(S-N)\left(1-\phi \left(\rho_{m}R,m,r\right)\right)+N$$ (4)

In the formula: γ(r) is the semivariance value; *S* is the sill value, which in this study is set to the variance of the data samples; *N* is the nugget effect value, representing small-scale noise or measurement error, and in this study is set to 5% of the variance; *R* is the range parameter of the semivariance; $\rho_{m}$ is a proportionality constant dependent on the order *m* of the spherical function.

In the Leapfrog software, the support radius *c* in this study is set to 1.5 times the basic survey spacing; the regularization parameter *λ* is set to 1 × 10⁻⁶ to balance the fitting accuracy and the stability of the solution; the hierarchical partitioning depth is automatically optimized based on the data volume; the truncation threshold is set to 1 × 10⁻⁴ to ensure the computational efficiency of the far-field fast multipole approximation. Under this configuration, the complexity of a single matrix-vector multiplication is *O*(*N*), and if the number of iterations is k, the total complexity of the construction phase is:


O(NlogN)+O(kN)=O(NlogN+kN) (5)

k is typically a constant, so the complexity of constructing the algorithm simplifies to *O*(*N*log*N*).

To determine the weight coefficients *w*_*i*_ and the first-order polynomial coefficients *a*_*i*_, the orthogonal condition (6) and the interpolation constraint condition (7) must be satisfied.


$$\sum_{i=1}^{n}w_{i}=\sum_{i=1}^{n}w_{i}\overset{x}{\underset{i}{q}}=\sum_{i=1}^{n}w_{i}\overset{y}{\underset{i}{q}}=\sum_{i=1}^{n}w_{i}\overset{z}{\underset{i}{q}}=0$$ (6)

$$f\left(q_{i}\right)=P\left(q_{i}\right)+\sum_{i=1}^{n}w_{i}\phi \left(q_{i}-q_{j}\right)=h_{i}$$ (7)

where: *j* = 1, 2, …, *n*.

Let $\phi_{i,j}=\phi \left(|\;q_{i}-q_{j}|\;\right)$, and from equations (6) and (7), we can obtain:


$$\left[\begin{array}{c} \phi_{11}\;\phi_{12}\;\cdots \;\phi_{1n}\;1\;\overset{x}{\underset{1}{q}}\;\overset{y}{\underset{1}{q}}\;\overset{z}{\underset{1}{q}}\\ \phi_{21}\;\phi_{22}\;\cdots \;\phi_{2n}\;1\;\overset{x}{\underset{2}{q}}\;\overset{y}{\underset{2}{q}}\;\overset{z}{\underset{2}{q}}\\ \vdots \;\vdots \;\vdots \;\vdots \;\vdots \;\vdots \;\vdots \;\vdots \\ \phi_{n1}\;\phi_{n2}\;\cdots \;\phi_{nn}\;1\;\overset{x}{\underset{n}{q}}\;\overset{y}{\underset{n}{q}}\;\overset{z}{\underset{n}{q}}\\ 1\;1\;\cdots \;1\;0\;0\;0\;0\\ \overset{x}{\underset{1}{q}}\;\overset{x}{\underset{2}{q}}\;\cdots \;\;\;\overset{x}{\underset{n}{q}}\;0\;0\;0\;0\\ \overset{y}{\underset{1}{q}}\;\overset{y}{\underset{2}{q}}\;\cdots \;\overset{y}{\underset{n}{q}}\;0\;0\;0\;\;\;0\\ \overset{z}{\underset{1}{q}}\;\overset{z}{\underset{2}{q}}\;\cdots \;\overset{z}{\underset{n}{q}}\;0\;0\;\;\;0\;0 \end{array}][\begin{array}{c} w_{1}\\ w_{2}\\ \vdots \\ w_{n}\\ a_{0}\\ a_{1}\\ a_{2}\\ a_{3} \end{array}]=[\begin{array}{c} h_{1}\\ h_{2}\\ \vdots \\ h_{n}\\ 0\\ 0\\ 0\\ 0 \end{array}\right]$$ (8)

Equation (8) can be simplified as follows:


$$\left(\begin{array}{c} \Phi \;Q\\ Q^{T}\;0 \end{array})(\begin{array}{c} W\\ A \end{array})=(\begin{array}{c} H\\ 0 \end{array}\right)$$ (9)

In the formula, $\Phi =[\begin{array}{c} \phi_{11}\;\phi_{12}\;\cdots \;\phi_{1n}\\ \phi_{21}\;\phi_{22}\;\cdots \;\phi_{2n}\\ \vdots \;\vdots \;\vdots \;\vdots \\ \phi_{n1}\;\phi_{n2}\;\cdots \;\phi_{nn} \end{array}]$, $Q=[\begin{array}{c} 1\;\overset{x}{\underset{1}{q}}\;\overset{y}{\underset{1}{q}}\;\overset{z}{\underset{1}{q}}\\ 1\;\overset{x}{\underset{2}{q}}\;\overset{y}{\underset{2}{q}}\;\overset{z}{\underset{2}{q}}\\ \vdots \;\vdots \;\vdots \;\vdots \\ 1\;\overset{x}{\underset{n}{q}}\;\overset{y}{\underset{n}{q}}\;\overset{z}{\underset{n}{q}} \end{array}]$, $W=[\begin{array}{c} w_{1}\\ w_{2}\\ \vdots \\ w_{n} \end{array}]$, $A=[\begin{array}{c} a_{0}\\ a_{1}\\ a_{2}\\ a_{3} \end{array}]$, $H={[h_{0},h_{1},\ldots ,h_{n}]}^{T}$ (10)

Under typical conditions, the left-hand side of equation (9) is an invertible non-positive semi-definite matrix, and thus there is only one set of solutions (*w*_*1*_, *w*_*2*_, …, *w*_*n*_, *a*_*0*_, *a*_*1*_, *a*_*2*_, *a*_*3*_,). The polynomial coefficients obtained by solving equation (9) can be used to determine *P*(*r*), and this should not vary with different geological units.

FastRBF represents an improved version of RBF that focuses on algorithmic acceleration and computational efficiency optimization, delivering enhanced performance and accuracy when processing large-scale datasets [39–40]. FastRBF employs hierarchical spatial partitioning by decomposing the computational domain into multi-level subspaces and implementing approximation schemes for distant regions. This reduces computational complexity from *O*(*N²*) in conventional RBF to *O*(*N*log*N*), enabling efficient modeling with millions of scattered points [41]. FastRBF addresses the dense matrix generated by global RBF interpolation through localized support kernel functions or truncation strategies, combined with Krylov subspace iterative solvers (e.g., GMRES), significantly reducing memory footprint and computational load. Compared to RBF, FastRBF achieves a 10-100x speedup in interpolation [42]; On the other hand, FastRBF employs multi-scale kernel functions (e.g., hybrid compactly supported Gaussian and global linear kernels) with data-driven bandwidth optimization to balance local detail resolution and global trend fitting. Adaptive adjustment of regularization parameters effectively mitigates interference from data noise and outliers [41].

### 3.2. Modeling workflow

**Fig 3:**
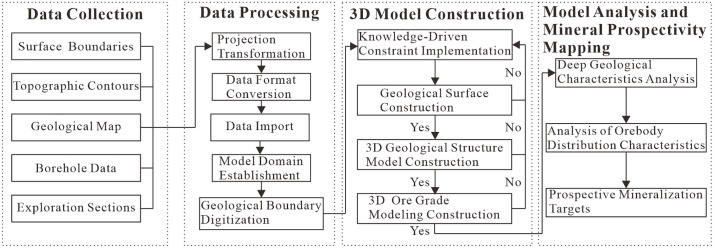
3D Geological modeling workflow.

The complex 3D geology of the study area makes it difficult to create accurate implicit models directly from borehole and exploration data. To address this challenge, our study introduces an optimized modeling workflow that integrates geological expertise as an interpretive constraint. Using the Songshugang Mining Area as a case study, we based this expertise on a comprehensive analysis of the region’s geological patterns and structural evolution. This knowledge-driven approach significantly enhances modeling accuracy and reliability, establishing a refined 3D geological modeling workflow (Fig 3). The implemented workflow comprises four critical phases: 1) Data collection, 2) Data processing, 3) Model construction, and 4) Model analysis with prospective mineralization targets prediction, detailed as follows:

(1) Data Collection. Prior to modeling, systematically collect the required input datasets, including surface boundaries, topographic contours, geological maps, borehole data, and exploration sections.
(2) Data Processing. This phase comprises format conversion, projection transformation, database integration, project boundary delineation, and georeferenced digitization of geological contacts.
(3) 3D Model Construction. Key procedures include expert knowledge constraint implementation, geological interface reconstruction, 3D structural framework modeling, and grade distribution modeling.
(4) Model Analysis and Mineral Prospectivity Mapping. The 3D structural and grade models enable comprehensive analysis of deep-seated geological characteristics and orebody distribution patterns, facilitating prediction of prospective mineralization targets at depth.

## 4. Modeling software and data processing

### 4.1. Modeling software introduction

The 3D geological modeling for this study was performed using Leapfrog Geo 2023.1, an industry-standard platform for implicit modeling. Its core strength is the FastRBF algorithm, which enables the rapid interpolation of diverse datasets to build high-precision models. These models effectively visualize complex geological features, including structural frameworks and orebody distribution. In addition to its primary algorithm, Leapfrog Geo enhances modeling efficiency through robust data processing, an intuitive interface, and powerful real-time updating and interactive editing functions. Its capabilities have led to its widespread adoption in fields such as mineral exploration, engineering geology, and hydrogeology.

### 4.2. Modeling data introduction

**Fig 4:**
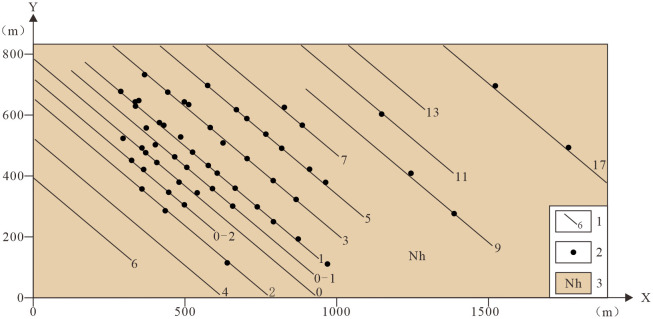
Boreholes and exploration section location map. 1. Exploration lines and labels; 2. Boreholes; 3. Nanhua System.

(1) Surface Boundaries. The modeling area extends 1.90 km (E-W) and 0.93 km (N–S), covering approximately 1.77 km². These boundaries define the lateral extents of the 3D model.

(2) Topographic Contours. Contour data from topographic maps serve as primary inputs for constructing the Digital Elevation Model (DEM). The generated DEM surface accurately represents terrain morphology, forming the upper boundary surface of the geological model.
(3) Geological Map. A 1:2,000-scale geological map incorporating 14 exploration sections and 57 borehole locations provides spatial constraints on geological unit boundaries in the 3D geological model, defining the volumetric extent of geological bodies.
(4) Borehole Data. A total of 57 boreholes were integrated, with depths ranging from 326.74 m to 1,000.25 m (mean 574.89 m). Boreholea are concentrated in the central and western sectors, with sparse coverage in the east. Datasets include collar coordinates, deviation surveys, stratigraphic logging, and assay analyses. Stratigraphic data supports 3D structural modeling, while assay data enables grade distribution modeling. Integration borehole data with exploration sections significantly enhances modeling accuracy [18,43–45].
(5) Exploration Sections. Fourteen exploration sections with NW-SE orientation were incorporated. Partial sections do not fully traverse the mining area. The central and western parts of the mining area have complex geological structures, with densely arranged exploration lines and profiles, while the eastern part has relatively simple geological structures and a sparser arrangement of exploration lines, resulting in a data distribution pattern characterized by “dense in the west and sparse in the east” (Fig 4). Accordingly, a differentiated modeling strategy was applied: virtual profiles were added in the structurally complex western area to better constrain 3D geometry, while no additional virtual profiles were required in the simpler eastern area.

### 4.3. Modeling data processing

(1) Projection Transformation. Surface boundaries, geological maps, exploration sections, and borehole collar coordinates underwent projection transformation to a unified 1:1,000 scale (for display purposes, not representing data precision) using CGCS 2000 ellipsoid parameters with Gauss-Krüger projection (3° zone 39).

(2) Data Format Conversion. Surface boundaries and topographic contours were converted to  .dxf format; raster geological maps and exploration sections to  .JPG format; borehole data were structured into relational tables: Collar, Survey, Interval, and Assay, linked by borehole IDs.
(3) Data Import. Import  .dxf format data directly into modeling software; spatially eoreferenced geological maps and exploration section maps in  .JPG format using the 3D coordinates (x, y, z) of four corner points; sequentially import drillhole collar tables, survey tables, interval tables, and assay tables to generate 3D drillhole trajectories. Perform error verification on imported data through multi-angle visualization inspection and software-based logical validation. Identified errors may be corrected either via direct edits or through source data amendments followed by reimportation.
(4) Model Domain Establishment. The model roof is defined by a DEM surface (peaking at 692.98 m), while the base constitutes a horizontal plane at −700 m elevation. Lateral boundaries are formed by vertical planes extending from the surface modeling delineation line.
(5) Geological Boundary Digitization. Perform interactive digitization of exploration sections based on geological unit distribution and contact relationships. ① Extract intersection points of geological interfaces from drillholes to obtain scattered point datasets; ② Digitize geological boundaries along exploration sections to capture interface traces. Strictly control node spacing during digitization, optimize boundary precision through precise node manipulation, ensure smooth and accurate representation of geological boundaries, and convert digitized boundaries into scattered point datasets.

## 5. 3D Geological model construction

### 5.1. Knowledge-driven constraint implementation

**Fig 5:**
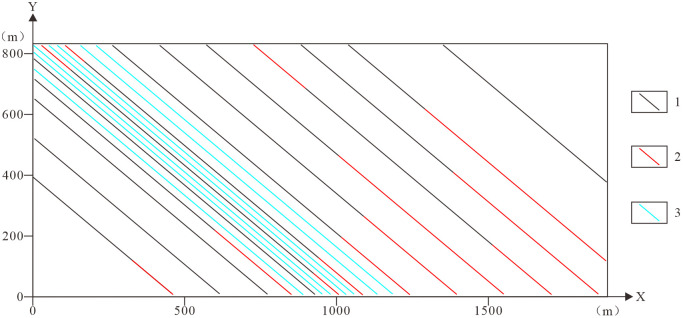
Distribution of extended exploration sections and interpolated virtual sections. 1. Original exploration sections; 2. Extended exploration sections; 3. Interpolated virtual sections.

(1) The deep rock mass in the study area exhibits a complex three-dimensional morphology with significant local variations, making it difficult to construct an accurate geological model using data-driven methods alone. Therefore, it is necessary to integrate expert knowledge to constrain the modeling process, thereby enhancing the accuracy and reliability of the model. Based on modeling data, regional geological background, and expert experience, the age relationships and spatial distribution characteristics of various geological bodies, as well as their three-dimensional morphology, can be clearly defined, providing a priori constraints for three-dimensional geological modeling.

(2) Expert knowledge constraints refer to the process of constraining and optimizing the construction of 3D geological models based on a deep understanding of regional geological characteristics by geological experts, integrating multiple sources of data such as geological maps, borehole data, and exploration line profiles. The key steps for implementing expert knowledge constraints in this study are as follows: first, constructing virtual geological cross-sections using expert knowledge; second, converting these virtual cross-sections into discrete 3D spatial points; and third, using these points as data sources to constrain the model, thereby optimizing the morphology of the 3D geological model. In situations where modeling data is insufficient, expert knowledge can leverage long-term accumulated geological research experience and regional geological patterns to make scientific inferences and supplement data in areas where information is missing or unclear. This study employs the following two methods to enhance model quality:

① In sections where geological bodies exhibit significant undulations, virtual cross-sections are obtained through equidistant interpolation based on existing exploration line profiles. Simultaneously, geological experts adjust the geological boundaries within these virtual cross-sections based on their understanding of the three-dimensional morphology of the geological bodies (Fig 5). The number of additional virtual cross-sections required should be determined by the complexity of the local geological bodies. For areas with complex morphology, a single virtual cross-section may not be sufficient to control the three-dimensional shape of the geological bodies, necessitating an appropriate increase in the number of cross-sections to supplement critical geological interface details, thereby significantly enhancing the accuracy and reliability of the modeling.

② When constructing geological interfaces using implicit function interpolation, the surfaces tend to extend automatically to the model boundaries. Because these boundaries are often poorly constrained by data, the automatically extended interfaces may deviate substantially from real geological conditions. To address this issue, the boundaries of existing exploration-line profiles can be expanded based on the variations in the direction and dip of the geological boundaries within the original exploration line profiles. This method ensures that the boundaries of the constructed geological interfaces are more consistent with actual geological conditions, effectively enhancing the continuity and geological rationality of the model boundary regions.

### 5.2. Geological surface construction

Modeling Units and Surface Typology. The model domain comprises eight primary units: Nanhua System (Nh), pegmatite-type Nb-Ta mineralization zone (ρ), greisenized granite-type Nb-Ta mineralization zone (γg), K-feldspathized granite-type Nb-Ta mineralization zone (γd), weak albitized granite subzone (γc), moderate albitized granite subzone (γb), strong albitized granite subzone (γa), and biotite granite zone (γ). Defined surface categories include DEM surfaces and geological interfaces.

**Fig 6:**
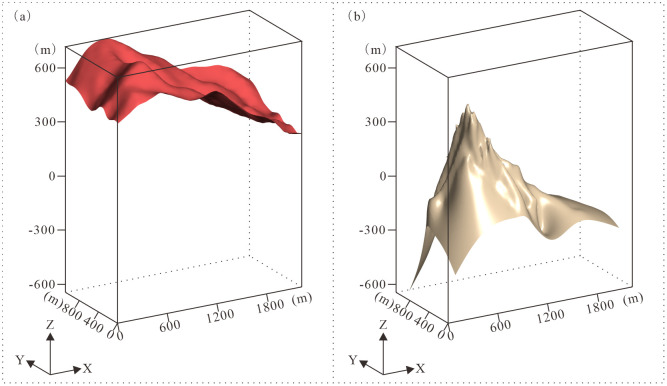
Display of DEM reference surface and nanhua system (Nh) geological interface. **(a)** DEM reference surface; **(b)** Geological interface of Nanhua System (Nh).

(1) DEM Surface Construction. Convert contour data with elevation attributes into scattered point datasets. Perform data validation to eliminate duplicate points. Generate 3D surfaces by triangulating the processed point set (Fig 6a). Where drillhole collar coordinates deviate from the DEM surface, spatially adjust triangulation nodes to ensure correspondence with actual collar positions.
(2) Geological Interface Construction. Digitize boundaries from exploration sections, densify nodes along digitized traces, and convert boundary data into scattered point datasets. Spatially interpolate these point datasets combined with borehole interval data through triangulation to construct geological interfaces (Fig 6b).

Refining Complex Geological Interfaces. Geological interfaces built solely from exploration sections and borehole data are often inaccurate for two reasons: (1) protracted exploration cycles resulting in section drafting based predominantly on drillhole data along individual lines, lacking comprehensive 3D geological continuity analysis that causes topological inconsistencies between adjacent sections; (2) uneven and sparse data distribution yielding inadequate constraint density for interface geometry. To correct these inaccuracies, we employ an integrated analysis that incorporates multiple sources of information: existing sections, borehole data, local structural knowledge, regional geological principles, and other site-specific features. Enhance constraint density through strategic insertion of virtual sections between existing exploration lines and extension of section coverage. Following expert-guided interpretive digitization of new boundaries on virtual/extended sections, fuse these with original data to reconstruct updated geological interfaces, completing the initial update cycle.

**Fig 7:**
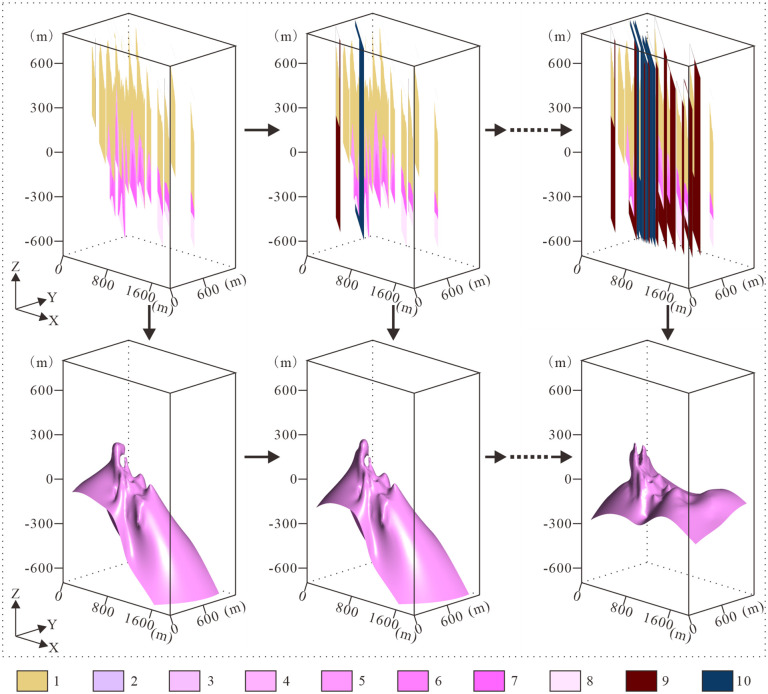
Schematic workflow of knowledge-constrained geological interface construction. 1. Nanhua System (Nh); 2. Pegmatite-type Nb-Ta mineralization zone (ρ); 3. Greisenized granite-type Nb-Ta mineralization zone (γg); 4. K-feldspathized granite-type Nb-Ta mineralization zone (γd); 5. Weak albitized granite subzone (γc); 6. Moderate albitized granite subzone (γb); 7. Strong albitized granite subzone (γa); 8. Biotite granite zone (γ); 9. Extended exploration sections; 10. Interpolated virtual sections.

To ensure that the constructed geological interfaces align with actual geological conditions, it is necessary to validate the updated geological interfaces. If discrepancies are found between the geological interfaces and the actual geological conditions, the exploration line profiles, virtual geological profiles, and extended exploration line profiles should be revised based on expert knowledge, and the geological interfaces should be updated again. The iteration can be terminated when the geological structures, shapes of geological bodies, and other key geological elements reflected in the model in three-dimensional space are consistent with objective geological data and expert judgment. Through repeated “validation-correction” iterative optimization processes, a geological interface that closely matches the actual conditions can ultimately be obtained (Fig 7).

### 5.3. 3D Geological structure model construction

First, an initial geological model was constructed by sequentially truncating a base volume. This process was guided by a chronological sequence of all geological interfaces (from youngest to oldest), determined from their spatial and cross-cutting relationships. During this stage, the appearance of any voids within the model served as a key validation check, signaling an incorrect chronological sequence that required immediate readjustment.

**Fig 8:**
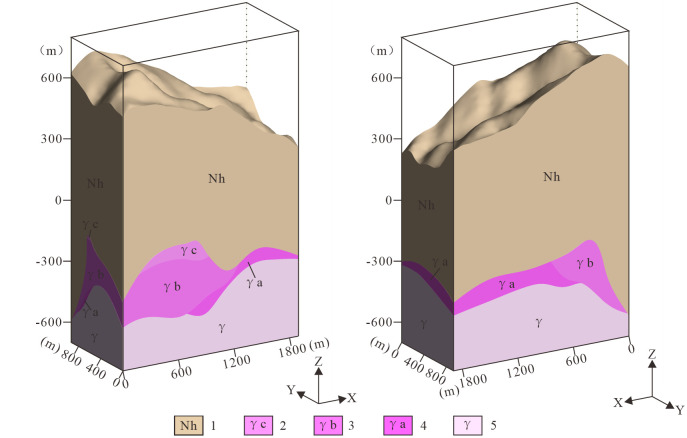
Schematic diagram of the 3D geological structure model. 1. Nanhua System (Nh); 2. Weakly Albitized Granite Subzone (γc); 3. Moderately Albitized Granite Subzone (γb); 4. Strongly Albitized Granite Subzone (γa); 5. Biotite Granite Zone (γ).

**Fig 9:**
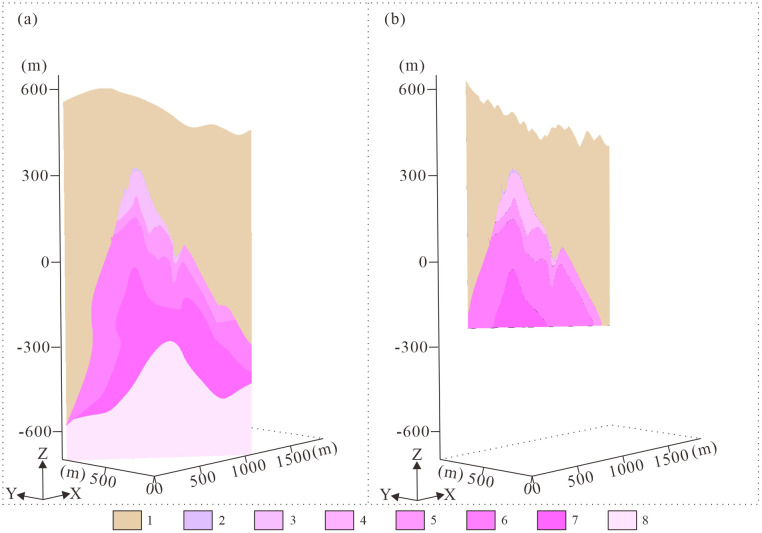
Comparison of model cross-section and actual profile. **(a)** Exploration Line 1 Model Slice; **(b)** Profile of Exploration Line 1. 1. Nanhua System (Nh); 2. Pegmatitic niobium-tantalum mineralization zone (ρ); 3. Greisenized granite-hosted niobium-tantalum mineralization zone (γg); 4. Potassic-altered granite-hosted niobium-tantalum mineralization zone (γd); 5. Weakly albitized granite subzone (γc); 6. Moderately albitized granite subzone (γb); 7. Intensely albitized granite subzone (γa); 8.Biotite granite zone (γ).

After the model is constructed, its consistency with the actual geological conditions must be verified. If discrepancies are identified, expert knowledge should be integrated to revise the existing exploration line cross-sections (including virtual geological cross-sections and extended exploration line cross-sections). If necessary, additional virtual geological cross-sections can be added, and the geological interfaces should be updated accordingly. Through this iterative optimization process based on expert knowledge, the model should be refined until it closely matches the existing boreholes, measured cross-sections, and other modeling data. The model should also ensure smooth transitions between rock bodies and geological interfaces, and its overall morphology should conform to regional geological patterns and align with the understanding of geological experts (Fig 8). The accuracy of the model can be validated by comparing model slices with existing actual cross-sections and boreholes (Fig 9). The final model should be spatially consistent with all existing boreholes and cross-section data.

### 5.4 3D Ore grade modeling construction

**Fig 10:**
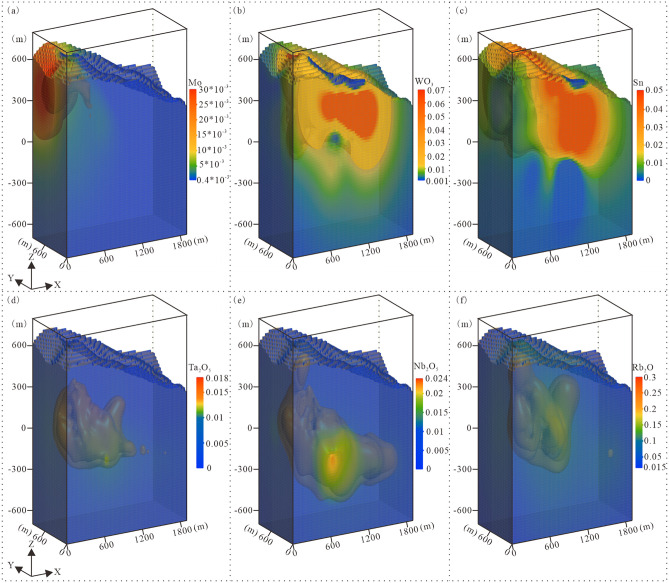
Schematic diagrams of the 3D ore grade models. **(a)** Mo grade model; **(b)** WO₃ grade model; **(c)** Sn grade model; **(d)** Ta₂O₅ grade model; **(e)** Nb₂O_5_ grade model; **(f)** Rb₂O grade model.

The borehole assay data in the modeling domain primarily include tungsten trioxide (WO₃), tin (Sn), molybdenum (Mo), tantalum pentoxide (Ta₂O₅), niobium pentoxide (Nb₂O₅), and rubidium oxide (Rb₂O). Utilizing the 3D geological structure model as a geometric constraint framework, create an attribute-empty 3D orebody solid model. Employ the FastRBF function to interpolate ore block cells within this model based on grade data of individual oxides, ultimately constructing the 3D ore grade model. Conduct mathematical statistical analysis of oxide grade data within the model, establish grade classification ranges, and visualize the results through color-coded rendering (Fig 10).

## 6. Model analysis and application

### 6.1. Deep geological characteristics analysis

**Fig 11:**
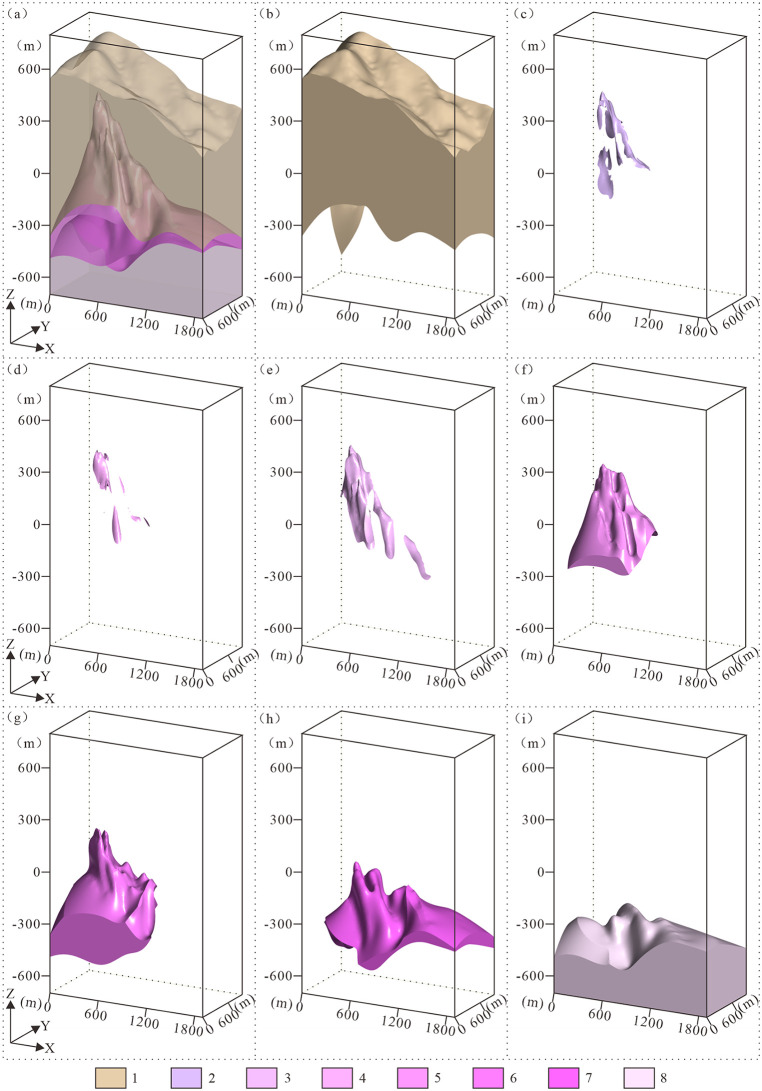
3D visualization of geological bodies. **(a)** 3D geological structure model; **(b)** Nanhua System (Nh) geobody; **(c)** Pegmatitic niobium-tantalum mineralization zone (ρ) geobody; **(d)** Greisenized granite-hosted niobium-tantalum mineralization zone (γg) geobody; **(e)** Potassic-altered granite-hosted niobium-tantalum mineralization zone (γd) geobody; **(f)** Weakly albitized granite subzone (γc) geobody; **(g)** Moderately albitized granite subzone (γb) geobody; **(h)** Intensely albitized granite subzone (γa) geobody; **(i)** Biotite granite zone (γ) geobody. 1. Nanhua System (Nh); 2. Pegmatitic niobium-tantalum mineralization zone (ρ); 3. Greisenized granite-hosted niobium-tantalum mineralization zone (γg); 4. Potassic-altered granite-hosted niobium-tantalum mineralization zone (γd); 5. Weakly albitized granite subzone (γc); 6. Moderately albitized granite subzone (γb); 7. Intensely albitized granite subzone (γa); 8. Biotite granite zone (γ).

Within the study area, the following geological units develop sequentially from surface to subsurface: Nanhua System (Nh), Pegmatitic Nb-Ta Mineralization Zone (ρ), Greisenized Granite Nb-Ta Mineralization Zone (γg), Potassic-Altered Granite Nb-Ta Mineralization Zone (γd), Weakly Albitized Granite Subzone (γc), Moderately Albitized Granite Subzone (γb), Strongly Albitized Granite Subzone (γa), and Biotite Granite Zone (γ) (Fig 11). The subsurface rock mass exhibits distinct ore-type zoning, vertically from top to bottom, four natural ore types are developed: the Pegmatitic Nb-Ta Mineralization Zone (ρ), the Potassic-Altered Granite Nb-Ta Mineralization Zone (γd), the Greisenized Granite Nb-Ta Mineralization Zone (γg), and the Albitized Granite Nb-Ta Mineralization Zone. The Albitized Granite Nb-Ta Mineralization Zone is further subdivided based on alteration intensity and spatial position: the Weakly Albitized Granite Subzone (γc), the Moderately Albitized Granite Subzone (γb), the Strongly Albitized Granite Subzone (γa), and Unaltered Biotite Granite. The deposit is dominated by albitized granite-type ores, which form thick, stratoid bodies concentrated in the central and lower parts of the rock mass. Overlying these are greisenized granite-type ores, characterized by saddle-like distributions and zones of localized, intense greisenization. In contrast, the two remaining ore types are discontinuous and have poor lateral continuity, occurring in varied forms such as banded, thin-shelled, saddle-like, or massive bodies.

(1) The Nanhua System (Nh) occurs in the shallow subsurface, and exposed at the surface, spanning elevations from −601.33 m to 692.98 m.
(2) The Pegmatite-type Niobium-Tantalum Mineralization Zone (ρ) occupies the intrusion’s upper contact zone at 38.34–412.25 m elevations, exhibiting relatively consistent thickness (0–18 m).
(3) The Potassic-altered Granite-hosted Niobium-Tantalum Mineralization Zone (γd) is located at the southeastern apex of the pluton. Minor occurrences underlie pegmatitic mineralization zones, while the majority directly contacts the Nanhua System strata. This zone exhibits a thin-lens, extending 150–280 m downdip to the southeast and 30–160 m along strike northeast. It displays discontinuous distribution with irregular thickness variations ranging from 0 to 35 m, within an elevation interval of 397.12 to 261.13 m.
(4) The Greisenized Granite-hosted Niobium-Tantalum Mineralization Zone (γg) is stratigraphically constrained between the underlying albitized granite-hosted ores and the overlying pegmatitic and potassic-altered zones. Exhibiting a saddle-shaped morphology, this unit has a variable thickness of 15–170 m and occurs within an elevation range of 19.70 m to 395.64 m.
(5) The Weak Albitized Granite Subzone (γc) is located below the greisenized granite mineralization zone and above the moderately albitized mineralized zone. The ore body is thicker between exploration lines 2 and 3 and becomes thinner to the east. It exhibits a saddle-shaped or pseudo-stratiform distribution, with a wide distribution range. The elevation range of the ore body is from 299.32 to −259.00 m, and the maximum thickness controlled by a single engineering project is 247.28 m. The thickness is mainly concentrated between 40 and 80 m.
(6) The Moderately Albitized Granite Subzone (γb) underlies the weakly albitized mineralization zone and overlies the intensely albitized mineralization zone. The subzone contacts metamorphic rocks or overlying porphyritic granite along its northwestern and southeastern margins. Occurring at −312.00 to 189.40 m elevations, it demonstrates a maximum borehole intersection of 135.66 m, with predominant thicknesses concentrated between 40–180 m.
(7) The Strongly Sodic Plagioclase-altered Granite Subzone (γa) is located beneath the moderately sodic plagioclase-altered mineralized zone and represents the deepest mineralized rock exposed by drilling. The elevation range of the ore body is −25.33 to −330.76 m, with a maximum thickness of 207.36 m controlled by a single engineering project. The thickness is primarily concentrated between 65.61 and 153.16 m.
(8) The Biotite Granite (γ) forms the basal unit of the model, underlying the intensely albitized granite zone. The orebody occurs below approximately −230 m elevation.

### 6.2. Analysis of orebody distribution characteristics

**Fig 12:**
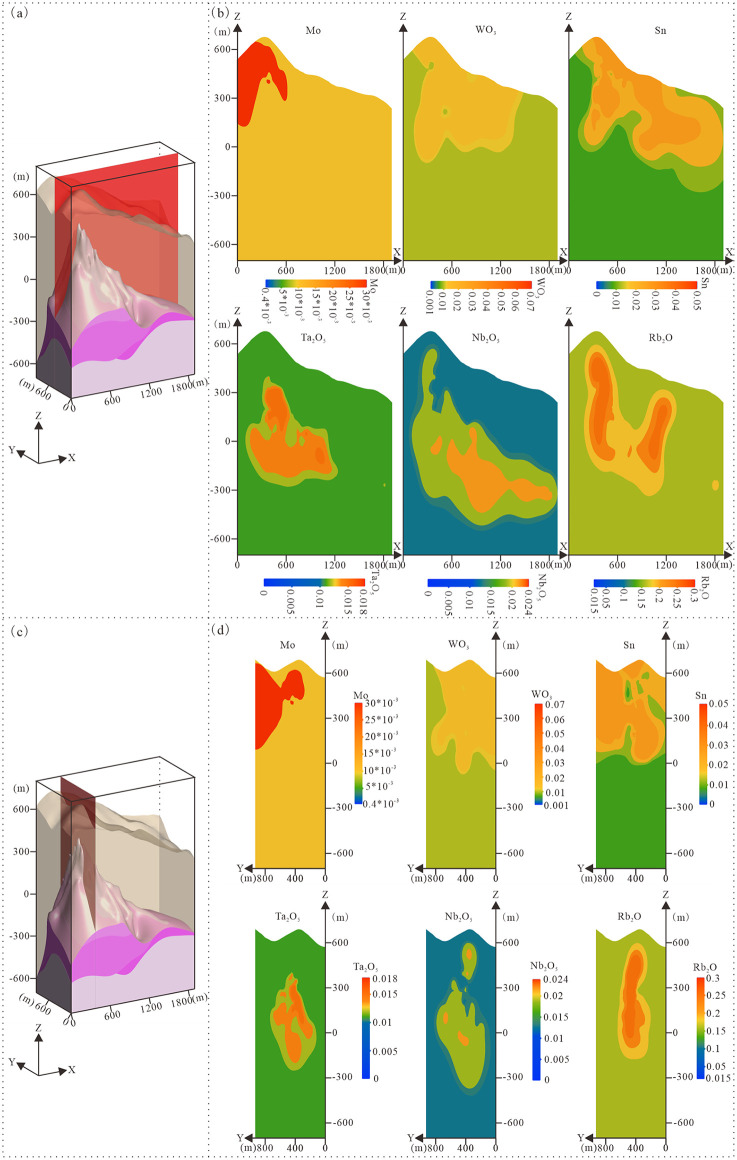
E-W and N-S section views of 3D grade model. **(a)** E-W section location diagram of 3D geological structure model; **(b)** Multi-element grade distribution section along E-W orientation; **(c)** N-S section location diagram of 3D geological structure model; **(d)** Multi-element grade distribution section along N-S orientation.

(1) Molybdenum, tungsten, and tin orebodies primarily occur within the Nanhua System strata, concentrated in the exocontact zone of the granite pluton (Figs 10 and 12). The granite underwent multistage evolution from magmatic crystallization to hydrothermal alteration, with greisenization and potassic alteration representing key metallogenic episodes for molybdenum mineralization. These alteration zones exhibit enriched Mo-W-Sn mineralization. Spatially, the deposit exhibits pronounced zonation, with grades peaking in the alteration centers and diminishing outwards. This pattern varies by metal: molybdenum orebodies, though limited in size, are concentrated in the northwestern sector, whereas tungsten and tin are enriched along the northern and southern margins, defining a depleted central core. In near-surface quartz veins, tungsten and tin orebodies demonstrate close paragenetic relationships. At depth, this association expands to include mineralization hosted in pegmatitic, potassic-altered, and greisenized granite. Mineralogically, tungsten principally occurs as wolframite and tin as cassiterite within quartz veins, typically co-crystallizing in these structures.

(2) Tantalum, niobium, and rubidium orebodies predominantly occur within the granite pluton, demonstrating peak mineralization enrichment in albitized and greisenized granites, with minor occurrences are observed in the exocontact zone (Figs 10 and 12). Orebodies typically exhibit disseminated distribution patterns, with morphology intrinsically controlled by pluton contacts and alteration zones. Spatially, grade distribution displays marked zonation, with maximum values in central alteration zones decreasing peripherally. Specifically, rubidium orebodies concentrate at the pluton apex, the granite-Nanhua System contact, and transitional domains between greisenized and albitized granites. Tantalum and niobium orebodies primarily distribute within the pluton, particularly along greisenized-albitized granite interfaces. Although greisenized granites yield higher Ta-Nb grades, limited development of such rocks renders albitized granites the principal host. Within these units, tantalum and niobium minerals occur as complex oxides-showing close spatial association but distinct mineralization characteristics-with rubidium orebodies demonstrating significant spatial correlation with Ta-Nb mineralization.

### 6.3. Prospective mineralization targets

**Fig 13:**
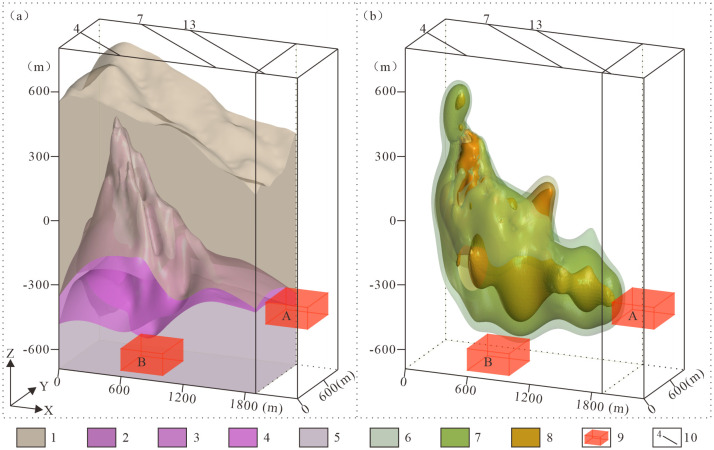
Predictive model of the songshugang Ta-Nb deposit. **(a)** 3D geological structure model; **(b)** 3D Ta-Nb grade model. 1. Nanhua System (Nh); 2. Weakly albitized granitic subzone (γc); 3. Moderately albitized granitic subzone (γb); 4. Strongly albitized granitic subzone (γa); 5. Biotite granitic zone (γ); 6. Ta > 0.01% and Nb > 0.015%; 7. Ta > 0.015% and Nb > 0.02%; 8. Ta > 0.018% and Nb > 0.024%; 9. Predicted favorable target zones; 10. Exploration line locations and numbers.

The tantalum-niobium orebodies in the Songshugang mining area are primarily controlled by hydrothermal alteration, with their spatial distribution closely linked to the 3D morphology of the granitic body and alteration zones. The 3D geological model (Fig 13) reveals significant enrichment of tantalum-niobium mineralization within granitic alteration zones. The orebodies are found primarily along the upper and lower contacts of the albitized granite, and notably, the eastward extension of this enrichment zone remains undrilled, indicating substantial deep-seated prospecting potential.

Based on the three-dimensional geological model, two new favorable mineral exploration targets have been delineated (Fig 13). Target Zone A, located east of Exploration Line 13 (elevations −300 m to −400 m), this is an unexplored area adjacent to the Lingshan pluton. It has been prioritized for systematic new exploration. Target Zone B, located between Exploration Lines 4 and 7 (elevations −600 m to −700 m), this zone represents a potential deep extension of known orebodies. This target is supported by our model, which indicates that Ta-Nb mineralization continues downward beyond the limits of current deep-level exploration. Both predicted zones demonstrate promising mineralization prospects and are designated as priority targets for the next exploration phase.

## 7. Conclusions and discussion

### 7.1. Conclusions

This study utilized the Leapfrog Geo platform to integrate multisource data, including borehole logs and exploration line profiles. Employing the FastRBF algorithm, we constructed expert knowledge-constrained 3D geological structure and grade models of the Songshugang Ta-Nb mining area in Hengfeng, Jiangxi Province. Systematic analysis of deep geological structures and Ta-Nb mineralization prediction yielded the following key findings:

(1) For the complex geological setting of the Songshugang mining area in Hengfeng, Jiangxi, an innovative expert knowledge-constrained implicit 3D modeling approach was developed. An iterative “modeling-verification-modification” workflow was employed to produce structural and grade models that are highly consistent with real-world geology, effectively overcoming the challenges of representing complex geological structures.
(2) Analysis of the 3D geological structure and grade models revealed two primary zones of significant Ta-Nb enrichment: the contact between albitized granite and the overlying K-feldspathized/greisenized granite, and its interface with the underlying biotite granite. Based on these findings, we have delineated two high-priority exploration targets, providing a robust geological basis for future prospecting efforts.

### 7.2. Discussion

While this study has yielded significant findings, certain aspects require further refinement:

(1) In the southwestern and northeastern sectors of the mining area, limited borehole density and sparse exploration line coverage constrained expert knowledge application, introducing modeling uncertainties. Subsequent model refinement requires supplemental data acquisition to enhance model accuracy.
(2) Although the expert knowledge-constrained implicit 3D geological modeling approach significantly enhanced modeling accuracy for complex structures, divergent geological interpretations among experts-regarding regional tectonic evolution, geological contact relationships, and deep 3D geometry-may lead to inconsistent constraint criteria. Future work could integrate machine learning algorithms to optimize expert knowledge constraints.
(3) Although the currently delineated favorable prospecting targets based on the 3D geological model are geologically constrained, they require engineering verification. Particularly given the complex deep-seated tectonic evolution and multiphase mineralization in the mining area, unidentified ore-controlling factors may impact prediction reliability.

## References

[1] Xiang ZL, Wang Y, Wang RH, Liu YF, Liu SX. 3D geological modeling and visualization process of mines based on borehole data. Geol Explor. 2009;45(1):75–81.

[2] Wu ZC. Three-dimensional geological modelling and deep ore-forming conditions analysis of uranium deposits in the Xiangshan volcanic basin, Jiangxi province. Nanchang: East China University of Technology; 2020.

[3] Xue T, Bao XS, Zhu XD, Huang X. Attribute modeling constrained by multi-source data-based 3D geological structural model: a case study in Tongzhou District, Beijing. Earth Sci Front. 2023;30(3):529–36. doi: 10.13745/j.esf.sf.2022.9.6

[4] Wu ZC, Zheng X, Zhang YY, Luo JQ, Hou MQ. Technological methods of build fault plane based on digital geological mapping data. J Liaoning Tech Univ Nat Sci. 2015;34(11):1264–70. doi: 10.11956/j.issn.1008-0562.2015.11.010

[5] Yu JJ, Wang GC, Xu YX, Guo JS, Chen XJ, Yang W. Constraining deep geological structures in three-dimensional geological mapping of complicated orogenic belts: a case study from Karamay Region, Western Junggar. Earth Sci J China Univ Geosci. 2015;40(3):407–18. doi: 10.3799/dqkx.2015.032

[6] Wu ZC, Guo FS, Jiang YB, Luo JQ, Hou MQ. Methods of three-dimensional geological modeling based on geological sections. Geol Explor. 2016;52(02):363–75. doi: 10.13712/j.cnki.dzykt.2016.02.020

[7] Zhao YL, Liu LM, Hu RG. 3D geological modeling based on the occurrence of geological bodies and data characteristics: a case study from Yueshan ore field, Anhui. J Guilin Univ Technol. 2018;38(04):752–60. doi: 10.3969/j.issn.1674-9057.2018.04.018

[8] Zhao YL, Ord A, Tang JR, Hu RG, Yang QJ, Wang JC, et al. Study on modelling method of intersecting faults basedon surface and occurrence data. Geotect Metallog. 2023; 47(06): 1256–66. doi: 10.16539/j.ddgzyckx.2023.01.305

[9] Li ZL, Wu CL, Zhang XL, Weng ZP, Liu G, Zhang ZT. Discussion on dynamic orcbody modeling with geological science big data. Bull Geol Sci Technol. 2020;39(04):59–68. doi: 10.19509/j.cnki.dzkq.2020.0408

[10] Liu ZB, Zhang JQ, Du XF, Guo JT. Implicit 3D integrated modeling of complex geological structures in mining areas. J Northeast Univ Nat Sci. 2024;45(09):1317–25. doi: 10.12068/j.issn.1005-3026.2024.09.013

[11] Wang XH, Liu ZB, Zhang DL, Ma L, Chen BH, Lei XR. A TIN-GTP algorithm-based technique for 3D geological modeling of coal mines. Coal Geol Explor. 2024;52(09):41−48. doi: 10.12363/issn.1001-1986.24.04.0222

[12] Guo J, Wang X, Wang J, Dai X, Wu L, Li C, et al. Three-dimensional geological modeling and spatial analysis from geotechnical borehole data using an implicit surface and marching tetrahedra algorithm. Eng Geol. 2021;284:106047. doi: 10.1016/j.enggeo.2021.106047

[13] Zhong DY, Wang LG. Implicit and automatic modeling method for complex coal seam geological bodies integrating fault potential fields. J China Coal Soc. 2025;50(03):1705−1716. doi: 10.13225/j.cnki.jccs.2024.0969

[14] Ma HB, Guo JT. Study on facial-volumetric mixed 3D geological modeling based on sections. Metalmine. 2007;7:50–2.

[15] Guo FS, Wu ZC, Li X, Zhang WL, Zeng WL, Lin ZY. The 3D geological modeling of Xiangshan volcanic basinin Jiangxi province. Geol Bull China. 2018;37(2/3):421–34.

[16] Guo JT, Liu YH, Han YF, Wang XL. Implicit 3D geological modeling method for borehole data based on machine learning. J Northeast Univ Nat Sci. 2019;40(9):1337–42. doi: 10.12068/j.issn.1005-3026.2019.09.021

[17] Wu ZC, Guo FS, Zhang WL, Ying YG, Zhou WP, Li C. 3D geological modeling based on multi-source data merging of Xiangshan volcanic basin in Le’an of Jiangxi. J Guilin Univ Technol. 2020;40(2):310–22. doi: 10.3969/j.issn.1674-9057.2020.02.008

[18] Lei CY, Liu ZX, Wen H, Fan M, Jiang HB, Wang B. Research on 3D geological modeling of complex geological body based on multi-source data and prior geological knowledge. Geol Rev. 2022;68(4):1393–411. doi: 10.16509/j.georeview.2022.06.141

[19] Wang LF, Liu XL, Xu K, Du LZ, Xu ZH, Zhang BY. Bayesian-MCMC (Markov chain Monte Carlo) based three-dimensional geological model optimization by data and knowledge fusion. Earth Sci. 2024;49(08):3056–70. doi: 10.3799/dqkx.2023.069

[20] Guo JT, Wu LX, Zhou WH. Automatic ore body implicit 3D modeling based on radial basis function surface. J China Coal Soc. 2016;41(08):2130–5. doi: 10.13225/j.cnki.jccs.2016.0688

[21] Fu JM, Hu MS, Fang F, Chu DP, Li H, Wan B. Complex orebody 3D modeling using radial basis function surface incorporating stacking integration strategy. Earth Sci. 2024;49(3):1165–76. doi: 10.3799/dqkx.2022.433

[22] Liao Z, Li M. Research on the 3D implicit potential field modeling method for urban underground space based on the open-source Gempy. Earth Sci Front. 2024;31(03):482–97. doi: 10.13745/j.esf.sf.2024.2.30

[23] Ma FL, Wu ZC, Liu PH, Li HL, Li B, Guo Z. Implicit 3D geological modeling of Julong’an uranium deposit in Xiangshan, Jiangxi Province. Uranium Geol. 2024;40(3):506–15. doi: 10.3969/j.issn.1000-0658.2024.40.045

[24] Gu H, Yang ZQ, Gao M, Tang XW, Wang DX, Liu KS, et al. Three-dimensional geological modeling and mineral prospectivity mapping in the Weishancheng gold-silver district, Henan, China. Earth Sci Front. 2024; 31(03): 245–59. doi: 10.13745/j.esf.sf.2023.6.8

[25] Wu ZC, Guo FS, Zheng X, Zhang YY, Luo JQ, Hou MQ. The technical methods of three-dimension geological modeling based on PRB data. Acta Geol Sin. 2015;89(7):1318–30.

[26] Li JM, Huang X, Shi WJ, Wang YJ, Cui K, Kong FS. Three-dimensional comprehensive model and deep prediction of the Jinqingding gold deposit, Muping-Rushan metallogenic belt, Shandong, China. Bull Geol Sci Technol. 2021;40(06):151–64. doi: 10.19509/j.cnki.dzkq.2021.0615

[27] Braga FCS, Rosiere CA, Santos JOS, Hagemann SG, Salles PV. Depicting the 3D geometry of ore bodies using implicit lithological modeling: an example from the Horto-Baratinha iron deposit, Guanhães block, MG. REM, Int Eng J. 2019;72(3):435–43. doi: 10.1590/0370-44672018720167

[28] Wang Q, Zou YH. Three-dimensional geological implicit surface reconstruction based on intermediate contour morphological interpolation. Bull Geol Sci Technol. 2023;42(05):293–300. doi: 10.19509/j.cnki.dzkq.tb20220003

[29] Liu T. Magmatic-hydrothermal evolution and Nb-Tamineralization in the Lingshan complex, Southeast China. Wuhan: China University of Geosciences; 2023.

[30] Huang DT. Evolving characteristics and related rare-metal metallogenesis of Lingshan rock body. Geol Prospecting. 2003;(4):35–40.

[31] Zhu ZY, Wang RC, Wang DH, Li JK, Ren JG. Metallogenic mechanism and prospecting direction of Songshugang granite-type niobium-tantalum deposit Jiangxi province. Acta Petrol Sin. 2024;40(09):2786–802. doi: 10.18654/1000-0569/2024.09.11

[32] Yuan LF, Wu ZC, Xu Z, Huang J, Li HL, Zhang ZQ. Remote sensing interpretation of surface linear-ring structures and wall-rock alteration in the Lingshan area, Shangrao City, Jiangxi Province, China: implications for mineral prospecting. Geocarto Int. 2025;40(1):2584954. doi: 10.1080/10106049.2025.2584954

[33] Lou FS, Xu Z, Huang H, Xiong YY. Geological characteristics and prospecting significance of low grade super large granite mica-type lithium deposits in Jiangxi province. J East China Univ Technol Nat Sci. 2023;46(5):425–36. doi: 10.3969/j.issn.1674-3504.2023.05.001

[34] Carr JC, Beatson RK, Mccallum BC, Fright WR, McLennan TJ, Mitchell TJ. Smooth surface reconstruction from noisy range data. ACM Graphite. 2003;3(1):119–26. doi: 10.1145/604471.604495

[35] Guo JT, Wu X, Wang JM, Zhuo WH, Li CL, Li FD. A regional integrated geological modeling method based on the implicitization of coons surface. Geogr Geo-Inf Sci. 2018;34(1):1–6. doi: 10.3969/j.issn.1672-0504.2018.01.001

[36] Zhong D, Wang L, Bi L, Jia M. Implicit modeling of complex orebody with constraints of geological rules. Trans Nonferrous Met Soc China. 2019;29(11):2392–9. doi: 10.1016/s1003-6326(19)65145-9

[37] Guo J, Wang J, Wu L, Liu C, Li C, Li F, et al. Explicit-implicit-integrated 3-D geological modelling approach: a case study of the Xianyan Demolition Volcano (Fujian, China). Tectonophysics. 2020;795:228648. doi: 10.1016/j.tecto.2020.228648

[38] Wang W, Zhou J, Wang SJ, Li XF. Research on three-dimensional modeling of stratablock based on radial basis function. Rock Soil Mech. 2012;33(03):939–44. doi: 10.16285/j.rsm.2012.03.041

[39] Minimo LG, Lagmay AMFA. 3D modeling of the Buhi debris avalanche deposit of Iriga Volcano, Philippines by integrating shallow-seismic reflection and geological data. J Volcanol Geotherm Res. 2016;319:106–23. doi: 10.1016/j.jvolgeores.2016.03.002

[40] Creus PK, Basson IJ, Koegelenberg CK, Ekkerd J, de Graaf PJH, Bester M, et al. 3D Fabric analysis of Venetia Mine, South Africa: using structural measurements and implicitly-modelled surfaces for improved pit slope design and risk management. J Afr Earth Sci. 2019;155:137–50. doi: 10.1016/j.jafrearsci.2019.04.009

[41] Carr JC, Beatson RK, Cherrie JB, Mitchell TJ, Fright WR, McCallum BC, et al. Reconstruction and representation of 3D objects with radial basis functions. In: Proceedings of the 28th annual conference on Computer graphics and interactive techniques. 2001. pp. 67–76. doi: 10.1145/383259.383266

[42] Beatson RK, Bui HQ. Mollification formulas and implicit smoothing. Adv Comput Math. 2006;27(2):125–49. doi: 10.1007/s10444-005-7512-3

[43] Lemon AM, Jones NL. Building solid models from boreholes and user-defined cross-sections. Comput Geosci. 2003;29(5):547–55. doi: 10.1016/s0098-3004(03)00051-7

[44] Zhu LF, Wu XC, Pan X. Mechanism and implementation of error correction for 3D strata model. Rock Soil Mech. 2006;2006(2):268–71. doi: 10.16285/j.rsm.2006.02.020

[45] Wang B, Lei CY, Liu ZX, Fan M, Wang XQ, Ye FZ. A geological 3D modeling method of comprehensive geological section for Chengdu. Sediment Geol Tethyan Geol. 2021;41(1):112–20. doi: 10.19826/j.cnki.1009-3850.2020.11003

